# Comparative Analysis of Pasture Composition: DNA Metabarcoding Versus Quadrat‐Based Botanical Surveys in Experimental Grassland Plots

**DOI:** 10.1002/ece3.71195

**Published:** 2025-03-27

**Authors:** Hannah Vallin, Helen Hipperson, Jan Titěra, Laura Jones, Mariecia Fraser

**Affiliations:** ^1^ Pwllpeiran Upland Research Centre Aberystwyth University Aberystwyth UK; ^2^ School of Biosciences University of Sheffield Sheffield UK; ^3^ Department of Biology and Ecology Technical University Liberec Czechia; ^4^ National Botanic Garden of Wales Llanarthne Carmarthenshire UK

**Keywords:** botanical survey, DNA metabarcoding, ecological monitoring, grassland biodiversity, species composition

## Abstract

DNA metabarcoding provides a scalable alternative to traditional botanical surveys, which are often time‐consuming and reliant on taxonomic expertise. Here, we compare DNA metabarcoding with quadrat‐based botanical surveys to assess plant species composition in experimental grassland plots under four defoliation regimes (continuous grazing, rotational grazing, frequent cutting and conservation cutting). Botanical surveys identified 16 taxa, while metabarcoding detected 25 taxa, including the dominant species *Holcus lanatus* and *Lolium perenne* . Despite detecting more taxa, there were some discrepancies in identification, with the sequence data only able to resolve some taxa at the genus level (e.g., *Agrostis* spp. instead of *Agrostis capillaris* ) and potential species misidentifications (e.g., *Cardaminopsis helleri* vs. *Cardamine flexuosa* ). However, both methods provided comparable results and revealed statistically significant differences in species composition between treatments, with higher diversity in cut versus grazed plots. The semi‐quantitative nature of metabarcoding limits its capacity to accurately reflect species abundance, posing challenges for ecological interpretations where precise quantification is required. However, it provides a broader view of biodiversity and can complement traditional methods, offering new opportunities for efficient biodiversity monitoring. The findings support the integration of DNA metabarcoding into biodiversity assessments, particularly when used alongside traditional techniques. Further refinement of bioinformatics tools and reference databases will enhance their accuracy and reliability, enabling more effective monitoring of grassland biodiversity and sustainable management practices. This study highlights DNA metabarcoding as a valuable tool for understanding plant community responses to management interventions.

## Graphical abstract

DNA metabarcoding offers a scalable alternative to traditional botanical surveys for monitoring plant community composition under different management regimes in experimental grassland plots. Comparing quadrat surveys and DNA metabarcoding, we found both methods identified key species and showed higher species diversity in cut versus grazed plots. This study supports the integration of DNA metabarcoding as a complementary approach, enhancing the efficiency and scope of biodiversity monitoring.

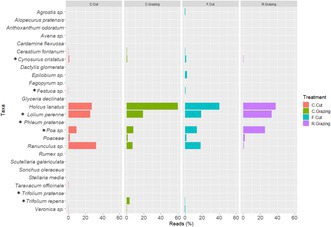

## 1 Introduction

Pressures from climate change and resource use continue to increase globally, threatening species with extinction and causing widespread shifts in plant communities, diversity and distribution (Ruppert et al. 2019). Grasslands, as globally significant ecosystems, are particularly vulnerable to these changes. They support high levels of biodiversity, provide critical ecosystem services such as carbon sequestration, and underpin sustainable livestock production. Accurately monitoring plant community composition in grasslands is essential for effective management yet remains challenging due to the limitations of traditional survey techniques.

Traditional methods to determine and monitor plant communities rely on visual botanical surveys to identify plant species via morphology‐based taxonomic practices (Mattia 2012). While reliable, such methods can be labor‐intensive and time‐consuming, requiring surveyors to have a high level of botanical expertise. However, there is a growing shortage of skilled taxonomic experts, limiting the ability to accurately characterize species composition and diversity (van der Heyde et al. 2020). These methods are also subject to observer bias and may require plants to be at specific growth stages to use morphological traits for identification, which is problematic since different species may flower at different times (De Mattia et al. 2012). These constraints can hinder large‐scale or long‐term biodiversity monitoring efforts, particularly in grasslands, where comprehensive and consistent data are crucial for effective livestock, conservation and management strategies.

In more recent years, technological advancements have paved the way for alternative DNA‐based methods such as DNA barcoding (identifying single species from individual specimens) and DNA metabarcoding (simultaneous identification of multiple species from a mixed sample) to be applied as means of determining botanical composition of habitats, and these are gaining popularity across various research disciplines, such as ecology and conservation biology, agricultural science and environmental monitoring (De Mattia et al. 2012; Leontidou et al. 2021; Banerjee et al. 2022). High‐throughput next‐generation sequencing has revolutionized DNA metabarcoding techniques, with the capability to process multiple sequences rapidly and cost‐effectively in parallel, often leading to higher taxonomic resolution, which is advantageous for large‐scale taxonomic investigations across diverse habitats and taxa (De Mattia et al. 2012; Fahner et al. 2016).

In comparison to traditional survey methods, DNA metabarcoding potentially offers additional benefits, such as the ability to identify plants throughout the growing season and the requirement for only a small amount of tissue for extraction and processing. While traditional surveys do not require physical sampling, DNA metabarcoding can significantly reduce the time spent in the field, which is particularly advantageous in challenging environments or adverse weather conditions. Additionally, samples collected for metabarcoding can be stored and processed later, providing a flexibility that traditional methods may lack. Early reviews, such as those by Taberlet et al. (2012), highlight that DNA metabarcoding could effectively assess plant diversity in complex environments, particularly in cases where visual identification is challenging, advocating for its use as a more comprehensive and scalable method compared to traditional surveys. Further research by Willerslev et al. (2014) highlighted the method's ability to detect a broad range of plant taxa, some of which are not easily identifiable through conventional means like pollen analysis.

Despite its advantages, DNA metabarcoding has limitations. These include PCR bias and the over‐representation of species with high chloroplast DNA content (Pornon et al. 2016); taxonomic resolution is constrained by more than just the reference database. The level of taxonomic distinction (e.g., species vs. genus) required depends on the ecological questions being addressed. While genus‐level identification might suffice in some studies, others may require species‐level resolution for precise ecological inferences (Banerjee et al. 2022; Braukmann et al. 2017; De Vere et al. 2012; Guo et al. 2022; Jones et al. 2021).

Recent advancements in DNA metabarcoding have demonstrated its potential for biodiversity monitoring across various ecosystems, but few studies have directly compared DNA metabarcoding with traditional botanical surveys of grasslands to evaluate the effectiveness of these methods in assessing plant biodiversity.

De Mattia et al. (2012) demonstrated the benefits of using a multi‐marker DNA barcoding approach to streamline plant species identification, significantly reducing both time and costs. However, their study did not incorporate metabarcoding or high‐throughput sequencing, which could further enhance its effectiveness. Other studies, like Yoccoz et al. (2012) and Duley et al. (2023), explored the use of soil eDNA for broader plant diversity assessments. While these studies found strong correlations between soil DNA and traditional above‐ground surveys, soil eDNA often captured more taxa, including species not visible above ground due to seasonal variations. Soil eDNA offers a reliable, non‐invasive, year‐round proxy for assessing plant diversity, providing an alternative to traditional plant surveys by detecting a broader range of species throughout different growth stages (Ariza et al. 2023; Carrasco‐Puga et al. 2021). Despite its advantages, soil eDNA might not accurately reflect above‐ground species composition, particularly in grazing systems, where available forage species are critical. Plant tissue‐based approaches, like the one employed in this study, provide a more accurate representation of forage species and resource availability, avoiding the challenges of PCR inhibition caused by substances like humic acid, which can affect soil DNA extractions (Uchii et al. 2019).

This study builds on these advancements by focusing on grass cuttings, which offer a more precise method for assessing species composition in grazing studies. Collectively, these studies underscore the growing importance of DNA metabarcoding in biodiversity research for species detection, dietary assessment, biomonitoring and habitat assessments, while acknowledging the need for continued refinements to overcome current limitations, with more research required to confirm its accuracy in comparison to traditional survey methods (Deiner et al. 2017; Leontidou et al. 2018; Fahner et al. 2016; De Mattia et al. 2012). These studies generally suggest that DNA metabarcoding has the potential to enhance and complement traditional botanical surveys, especially in detecting uncommon species and providing a more comprehensive assessment of plant communities. However, they also emphasize the need for the development of standardized protocols and up‐to‐date reference databases, which are essential for improving the capability to effectively integrate metabarcoding data into broader ecological analyses and conservation research (Jones et al. 2021).

To our knowledge, no such study has been conducted to directly assess the analysis of pasture composition using DNA metabarcoding of above‐ground vegetation samples versus traditional botanical surveys. This comparison is particularly novel because DNA metabarcoding, as an innovative application of environmental DNA, offers a way to overcome many past challenges associated with conventional methods, such as the need for extensive taxonomic expertise and time‐consuming fieldwork (Deiner et al. 2017). With grasslands facing increasing pressures from climate change, habitat loss and resource depletion (Banerjee et al. 2022), this study evaluates the reliability and effectiveness of DNA metabarcoding in monitoring biodiversity. As eDNA rapidly gains popularity in ecology and conservation due to its scalability and efficiency, this study addresses the growing demand to evaluate its reliability and effectiveness in monitoring grassland biodiversity against established methodologies.

Here, we explore whether DNA metabarcoding could be used as an alternative to a traditional quadrat‐based surveying methodology to characterize the botanical composition of mixed‐species pasture within a controlled experimental grassland plot subject to varying management regimes. The two methodologies were compared in terms of the quantification and composition of vegetation in a subset of plots established as part of a larger experiment exploring the interactive effects of defoliation type (cutting vs. grazing) and timing. The knowledge gained from this study has practical applications for real‐world global biodiversity challenges in grassland management and conservation, including advancing monitoring techniques that can inform evidence‐based management decisions, and supporting the development of predictive models for ecosystem conservation and livestock management. These insights will also aid in optimizing grazing regimes to enhance animal nutrition, while avoiding over‐exploitation and depletion of habitat resources, contributing to more sustainable agricultural practices.

## 2 Material and Methods

### 2.1 Experimental Design

Mixed swards were sown across four sites at Aberystwyth University's Pwllpeiran Upland Research Centre (70–340 m a.s.l.), which together form a long‐term ‘challenge gradient’ experimental resource. This gradient represents a series of sites at varying altitudes, each with differing environmental conditions such as soil type, moisture levels and temperature. These conditions mimic the challenges faced by grasslands under changing climatic and ecological pressures, allowing researchers to evaluate the performance and resilience of different grassland management strategies. The study reported here was performed on experimental grassland plots at the third site along the gradient (230 m a.s.l). In 2019, five replicates each of two sward mixtures were established as 28 m × 7 m plots (with plots at least 7 m apart) for a total of 10 plots. Mixture 1 was expected to be highly productive in favorable growing conditions, which include high soil fertility, consistent water availability and optimal temperatures, making it suitable for more productive lowland environments. This mixture was composed of 81% perennial ryegrass ( *Lolium perenne* ), 8% white clover ( *Trifolium repens* ), 6% timothy ( *Phleum pratense* ) and 5% red clover ( *Trifolium pratense* ). In contrast, Mixture 2 was expected to be tolerant of more marginal growing conditions and was made up of 45% perennial ryegrass, 13% meadow fescue ( *Festuca pratensis* ), 11% timothy, 9% creeping red fescue ( *Festuca rubra* ), 8% white clover, 5.5% smooth‐stalked meadow grass ( *Poa pratensis* ), 5% red clover, 2% lotus ( *Lotus corniculatus* ) and 1.5% crested dog'stail ( *Cynosurus cristatus* ).

After establishment, each plot was divided into four subplots, with one of four contrasting defoliation treatments applied to each subplot to assess the impact of defoliation type and frequency on botanical and chemical composition. The four management regimes were imposed annually from the start of April to the end of September: (1) continuous grazing (CG), where sheep had continuous access to pasture managed to a sward surface height of 5–6 cm (with all grazed subplots located within a single grazing enclosure); (2) rotational grazing (RG), where pasture was excluded from grazing for 2 weeks, and then access was given until the target sward height of 5–6 cm was reached or up to a maximum of 7 days, whichever was sooner; (3) frequent cutting (FC), where pastures were regularly cut to a height of 10 cm at 3‐week intervals and (4) conservation cutting (CC), where pastures were cut to a height of 10 cm at 6‐week intervals. During August 2022, all 40 subplots at the site were characterized using (i) a quadrat‐based surveying technique previously adopted on similar grassland (Pavlů et al. 2021) and (ii) DNA metabarcoding.

### 2.2 Botanical Survey

In July 2022, before the cutting treatments were carried out, the percentage cover of all plant species present was estimated visually in one randomly located quadrat measuring 0.5 m × 0.5 m within each sub‐plot (*n* = 40 in total). The surveying was conducted by an experienced botanist who had worked on similar grassland for some years. The nomenclature of the plant species followed the classification system described by Poland and Clement (2009).

### 2.3 DNA Metabarcoding

Immediately following the botanical surveys, the entire quadrat area was cut using portable cordless shears (Horner Razor, Lancashire, UK), and the bulked biomass was bagged individually and gently homogenized. The samples were frozen at −20°C immediately after sampling prior to freeze‐drying and milling through a 1 mm mesh, with thorough cleaning between samples with 80% ethanol to ensure no contamination.

DNA extractions (192) were carried out in duplicate with 0.03 g of material and the DNeasy 96 Plant Kit (Qiagen, USA, Valencia, CA) following the manufacturer's protocol with modifications to improve extraction success, as described by de Vere et al. (2012): adding 80 μL proteinase K (1 mg/mL) (Sigma) to 400 μL of the lysis buffer AP1 (Qiagen kit) and 1 μL of RNase A (Qiagen kit) to each sample before extending the first incubation phase to 1 h at 65°C, followed by tissue disruption with a TissueLyser II (Qiagen) with 3 mm tungsten beads. The final elution stage in the aqueous elution buffer (elution buffer) was also extended to 15 min. Vegetation samples were weighed out onto the extraction plate in a random order to avoid any bias in plate layout and the extraction process. DNA extraction was successful for all samples, as verified by running a subsample on an EPOCH microplate spectrophotometer and visualizing all extracts on a 1% agarose gel.

Illumina MiSeq paired‐end indexed amplicon libraries were created via a two‐step PCR protocol. Libraries were prepared for two DNA barcode regions, the complete second internal transcribed spacer of nuclear ribosomal DNA (ITS2), forward primer ITS2F: (5′‐ATGCGATACTTGGTGTGAAT‐3′) (Chen et al. 2009), reverse primer UniPlantR (5′CGHYTGAYYTGRGGTCDC‐3′) (Moorhouse‐Gann et al. 2018) and the Ribulose‐1,5‐bisphosphate carboxylase (*rbc*L) marker, forward primer *rbc*Laf: (5′TGTCACCACAAACAGAGACTAAAGC‐3′) (Kress and Erickson 2007), reverse primer *rbc*Lr506: (5′AGGGGACGACCATACTTGTTCA −3′) (De Vere et al. 2012).

Two‐stage PCR amplifications were performed in 20 μL reaction volumes. The first stage involved amplifying *rbc*L and ITS2 regions separately using specific primers tailed with adapter sequences. These markers were selected for their complementary strengths in plant DNA metabarcoding. The rbcL gene is a chloroplast marker commonly used for plant identification due to its broad taxonomic coverage, making it effective for identifying plant families and genera (Jones et al. 2021). Meanwhile, the ITS2 region, located in nuclear ribosomal DNA, provides higher taxonomic resolution at the species level due to its greater sequence variability (Moorhouse‐Gann et al. 2018). Combining these two markers allows for a more comprehensive and accurate characterization of plant communities, addressing both broad taxonomic representation (rbcL) and fine‐scale species‐level identification (ITS2) (Garnick et al. 2018; Jones et al. 2021; Moorhouse‐Gann et al. 2018). The reaction mix included 10 μL Phusion Hot Start II High‐Fidelity PCR Master Mix (Fisher Scientific), 0.8 μL of each primer (5 μM), 6.4 μL molecular biology grade H_2_O and 2 μL template DNA (1:10 diluted DNA extract). The *rbc*L thermal profile was 98°C for 30 s, 95°C for 5 min, then 35 cycles of 95°C for 30 s, 56°C for 30 s, 72°C for 1 min, with a final extension at 72°C for 10 min and 30°C for 1 min. For ITS2, conditions were 98°C for 30 s, 94°C for 10 min, followed by 34 cycles of 94°C for 30 s, 56°C for 30 s, 72°C for 45 s, with a final extension of 72°C for 10 min. PCR products were verified on a 1% agarose gel and purified using AMPure XP beads (Beckman Coulter) with a 0.6× bead:DNA ratio, eluting in 32.5 μL low Tris‐EDTA buffer.

The second PCR stage added unique index sequences and Illumina sequencing adapters using a 12.5 μL Phusion mix, 1 μL Fi5/Fi7 index Primer (0.2 μM), 6.5 μL H_2_O and 5 μL pooled PCR1 products. The cycling conditions were 98°C for 3 min, followed by 10 cycles of 98°C for 30 s, 55°C for 30 s, 72°C for 30 s and a final extension at 72°C for 5 min and 4°C for 10 min. Products were checked on a 1% agarose gel and purified with a 0.8× bead:DNA ratio, eluting in 27.5 μL PCR grade water. Negative controls were included to monitor for cross‐contamination. To determine the concentration of DNA, the purified products from the index PCR were quantified on a Qubit 3.0 Fluorometer using the high‐sensitivity dsDNA assay kit (Fisher Scientific) and were pooled in equimolar amounts to produce the final library for sequencing. Next‐generation DNA sequencing was carried out at the Centre for Genomic Research, University of Liverpool, using an Illumina MiSeq with a v3 2×300 bp sequencing kit.

Sequence data was processed using a custom Python script (https://github.com/colford/nbgw‐plant‐illumina‐pipeline). Raw reads were trimmed using Trimmomatic v0.33 (Bolger et al. 2014) and merged with FLASH v1.2.11 (Magoč and Salzberg 2011), retaining sequences over 350 bp (ITS2) and 450 bp (*rbc*L). Sequences were then demultiplexed, dereplicated and clustered at 100% identity using vsearch v2.3.2 (Rognes et al. 2016), with singletons removed from the dataset. Taxonomic assignment was performed against a curated reference library from the Barcode Wales and Barcode UK projects (De Vere et al. 2012; Jones et al. 2021), which covers 98% of UK native plants. Assignments were made based on the highest bit score from BLAST searches; species‐level assignments were confirmed if unique; otherwise, assignments were at genus or family levels depending on sequence similarity. Sequences with bitscore matches below 500 representing ambiguous high‐level taxonomic matches were excluded (Hawkins et al. 2015; De Vere et al. 2017). The taxonomic identification of the sequences was then manually verified to ensure accuracy, taking into account the botanical relevance from prior knowledge of the experimental site and the discriminatory power of each marker (Jones et al. 2021). The output was a final species matrix summarizing the total number of sequence reads found in each sample for each identified taxon.

The discriminatory power of the *rbc*L and ITS2 amplicons was evaluated, and a consensus taxonomy was established by integrating data from both markers, guided by botanical survey knowledge. This consensus approach was validated by non‐metric multidimensional scaling (NMDS) to visualize differences and similarities in community composition, demonstrating dataset similarity and supporting marker combination for enhanced taxonomic resolution. This approach ensured consistency and allowed for a more comprehensive representation of the taxonomic information and has also been demonstrated as an effective way to combine sequence data in previous studies (Chen et al. 2010; Hollingsworth et al. 2009; Jones et al. 2021; Lowe et al. 2022). Final taxonomic outputs were summarized in a species matrix reflecting sequence read counts per taxon, with proportions calculated to represent relative read abundance.

### 2.4 Statistical Analysis

The DNA metabarcoding data was converted to relative abundance for use in all analyses, while the botanical survey data retained its original abundance format for comparison. Non‐metric multidimensional scaling (nMDS) plots were generated using the vegan and ggplot2 (version 4.3.1) packages in R to visualize treatment dissimilarities based on plant taxa read proportions.

To examine the effect of treatment on species abundance and composition, multivariate analyses with generalized linear models (GLMs) were conducted using the mvabund package with a negative binomial distribution for over‐dispersed count data. Model diagnostics included box plots, Q‐Q residual plots for homoscedasticity checks, and ANOVA tests to assess species composition changes under different management treatments. Individual species effects were analysed using univariate tests with adjusted *p*‐values for dependencies among responses. Permutation‐based Mantel tests in PAST (version 4) evaluated correlations between DNA metabarcoding and botanical survey data using Bray–Curtis dissimilarity indices.

Shannon‐Weiner diversity indices were calculated followed by Shapiro–Wilk tests for normality. Post hoc Tukey's tests determined significant differences between treatments. Redundancy Analysis (RDA) explored relationships between species composition and treatment variables, with data normalized to species relative abundance and visualized through standard biplot ordination diagrams using the ggvegan package.

## 3 Results

### 3.1 Botanical Composition and Impacts of Management as Determined Using Quadrat‐Based Botanical Surveying

**FIGURE 1:**
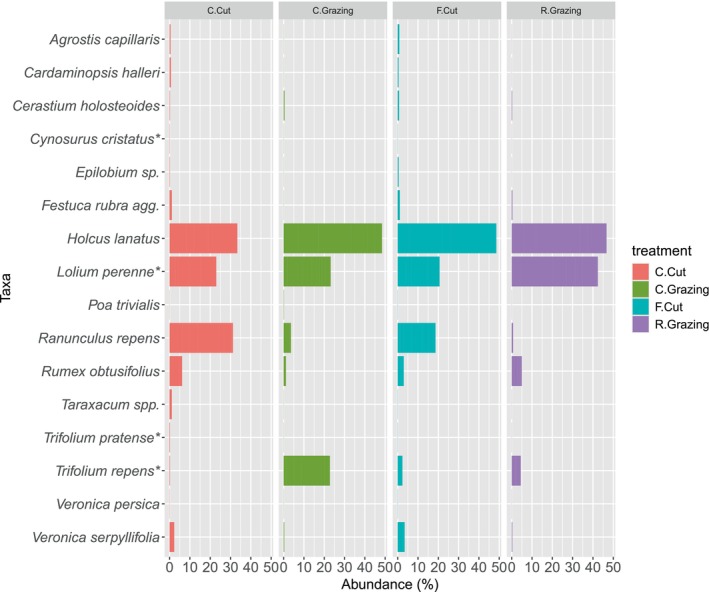
Taxa bar plot to show species identified (% abundance) across the grassland plot from botanical survey results for each management treatment, conservation cut (C.Cut), conservation grazing (C.Grazing), frequent cut (F.Cut) and rotational grazing (R.Grazing). (*) Indicates species that were originally sown in the mixture varieties.

**FIGURE 2:**
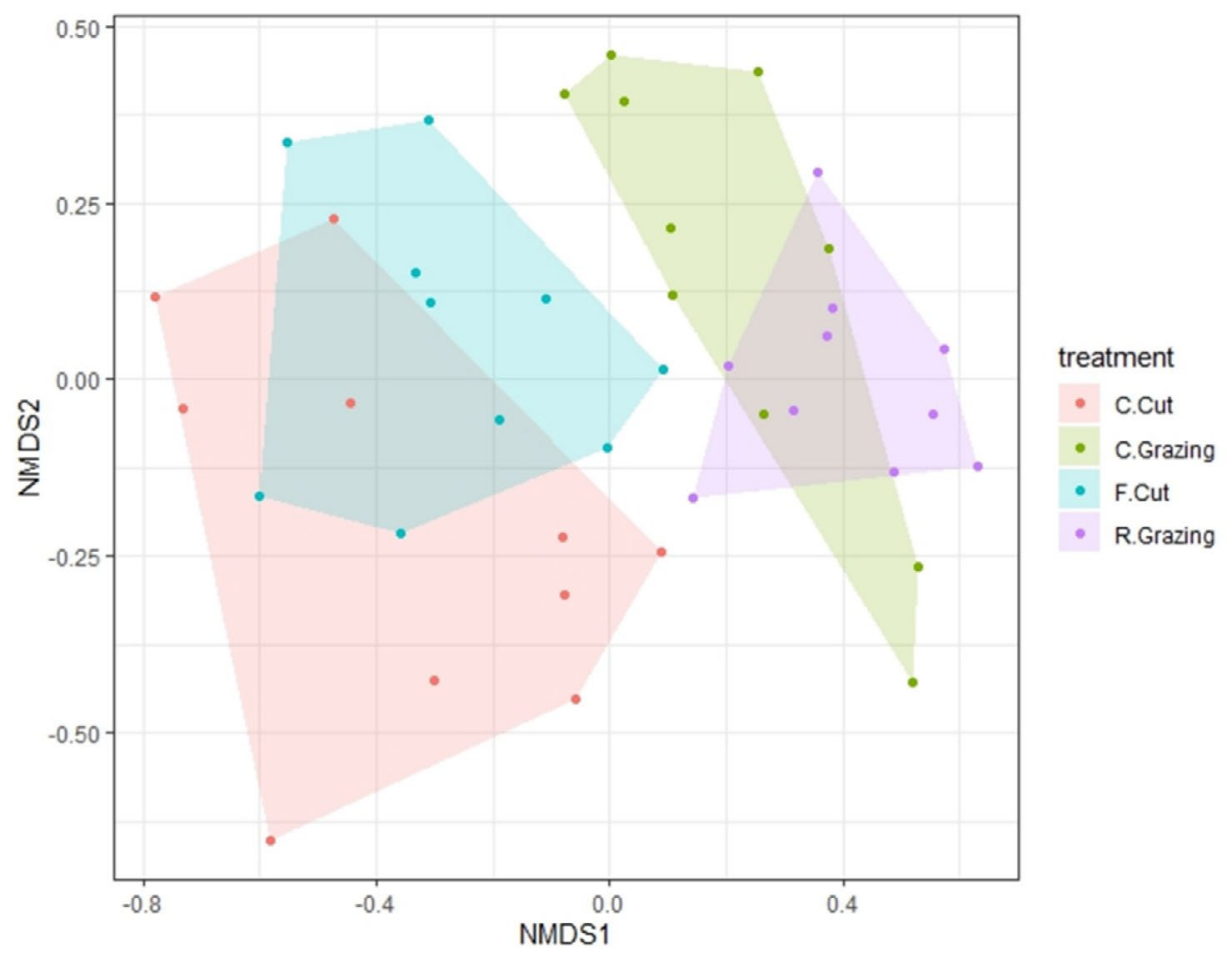
Non‐metric multidimensional scaling (nMDS) ordination plot using Bray–Curtis dissimilarity on relative abundance of botanical survey data. The four management treatments present are: Conservation cut (C.Cut), conservation grazing (C.Grazing), frequent cut (F.Cut) and rotational grazing (R.Grazing).

The vegetation survey detected a total of 16 taxa belonging to 8 orders, 9 families and 14 genera. The most frequently detected taxa in the survey plots were *Holcus lanatus, L* *. perenne* and *Ranunculus repens* (Figure 1), mirroring patterns seen in the metabarcoding results in the following section 3.2. However, the NMDS ordination plots visually show a greater separation between the cutting and grazing regimes (Figure 2).

The botanical survey dataset revealed significant differences in plant composition between the four treatment groups (LR_36_ = 174.7, *p* = 0.001) with a higher diversity in the cutting regimes compared to the grazing regimes. Following a significant ANOVA result, Tukey's test was used for pairwise comparisons, revealing significant differences among treatments (*p* = 0.005), though no significant effect was found for mixture variety (*p* = 0.065). This finding aligns with the results obtained from the metabarcoding dataset. However, there were more significant differences between treatments, particularly noticeable in the comparison between the cutting and grazing regimes (Table 1). 24.74% of the species data variance was accounted for between treatments (*R* ^2^ = 0.247; adjusted *R* ^2^ = 0.185), indicating a moderate impact of the treatment factor on the species data contributing to the observed diversity. This was further supported by conducting a permutation test, with a substantial proportion of variance in the species data explained by treatment as a predictor (Variance = 0.161, *F* = 4.334, *p* < 0.001).

**TABLE 1:** Pairwise comparisons between treatment groups to identify which specific groups are different from each other using Tukey's test and the botanical survey dataset.

Treatment	*p* adj	
C.Grazing‐C.Cut	0.009	**
F.Cut‐C.Cut	0.960	
R.Grazing‐C.Cut	0.004	**
F.Cut‐C.Grazing	0.032	*
R.Grazing‐C.Graz	0.989	
R.Grazing‐F.Cut	0.015	*
Mix Variety	*p* adj	
Landmark‐Century	0.650	

* Indicates a significant result.

**FIGURE 3:**
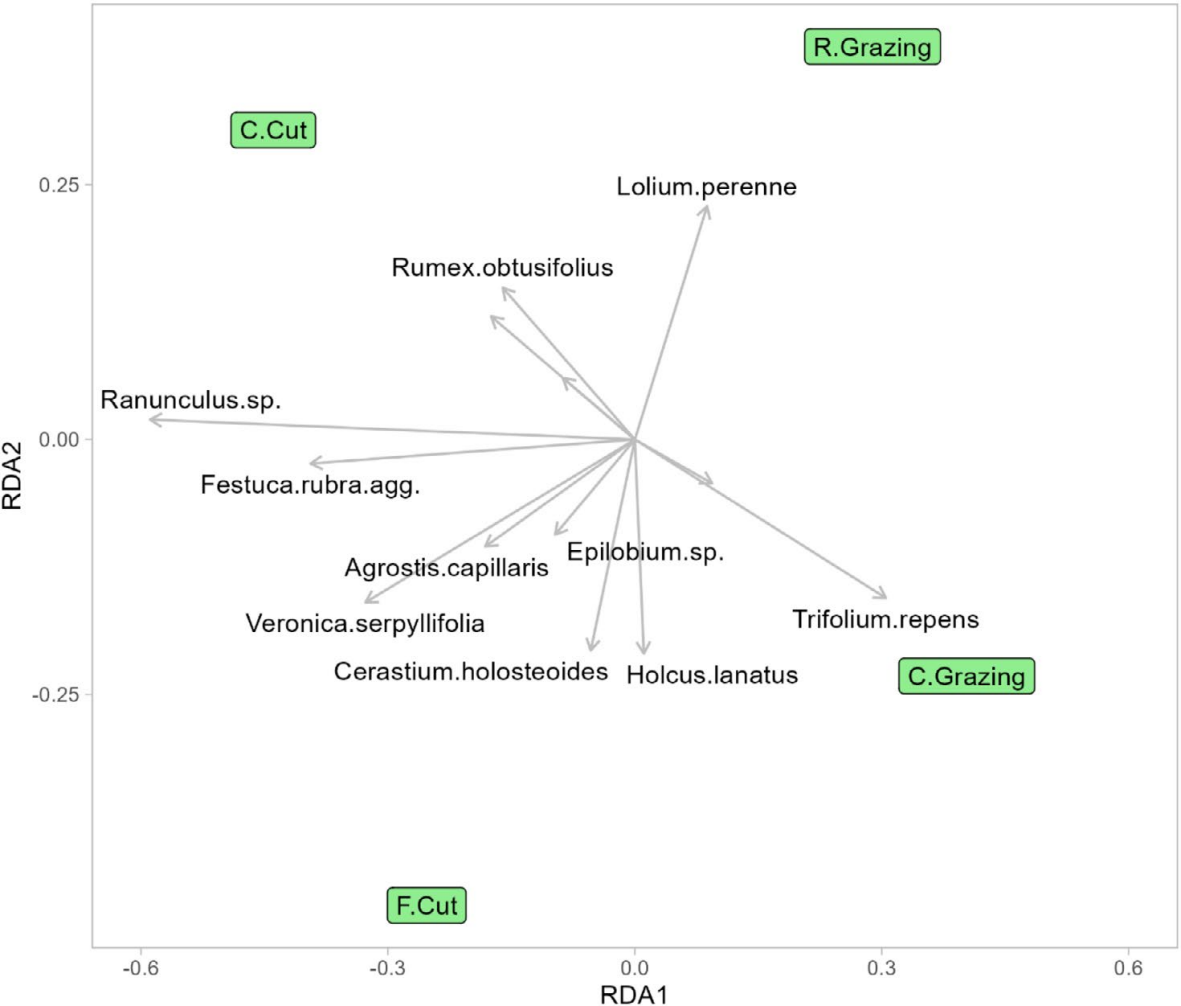
Ordination diagram representing the results of redundancy analysis (RDA) on the botanical survey dataset displaying the most frequently found plant species within each treatment. Rotational (R.) grazing, continuous (C.) grazing, frequent cutting (F.Cut), conservation cutting (C.Cut).

The ordination plot (Figure 3) reveals the extent of treatment impact on the species present. The two grazing regimes are characterised as being species‐poor in comparison to the cutting treatments, with *L. perenne* dominating the rotational grazing treatment and *T. repens*, the continuous grazing treatment. Conversely, the conservation cut and frequent cut treatments favored *Ranunculus* spp., *Rumex obtusifolius, F* *. rubra* , *Veronica serpyllifolia* and *Cerastium holosteoides* , consistent with patterns seen in the metabarcoding dataset in 3.2. Nonetheless, a distinct variation becomes apparent in the case of *H. lanatus* , where it appears to be more dominant within the continuous grazing treatment according to the metabarcoding dataset. Conversely, the botanical survey indicates its prevalence not only in the continuous grazing treatment but also in the frequent cutting treatment.

### 3.2 Botanical Composition and Impacts of Management as Characterized Using DNA Barcoding

DNA metabarcoding of 197 samples with *rbc*L and ITS2 returned a total of 13,963,284 reads, of which 7,709,023 remained after trimming, pairing and merging sequences. Unidentified reads were removed during data preprocessing to ensure that only high‐quality, taxonomically assignable sequences were included in downstream analyses. Less than 1% of reads were excluded from the ITS2 dataset, while fewer than 2% were removed from the rbcL dataset. After filtering to remove ambiguous sequences and singletons, 4,648,385 sequences remained as the final total for the combined markers to analyse. A total of 23 taxa were identified using ITS2, comprising 23 identified to family, 9 to genus and 14 to species, whilst a total of 25 taxa were identified using *rbc*L, 25 identified to family, 11 to genus and 14 to species. Both markers showed similar discriminatory power overall. However, while *rbc*L was able to identify Poaceae to the family level and other genera within Poaceae, ITS2 provided a more detailed resolution, distinguishing Poa at the genus level. This distinction highlights the complementary nature of the two markers, particularly in identifying species within complex families like Poaceae. All negative control samples had either no or very few sequences (< 1% of total reads) present and were removed from further analysis.

**FIGURE 4:**
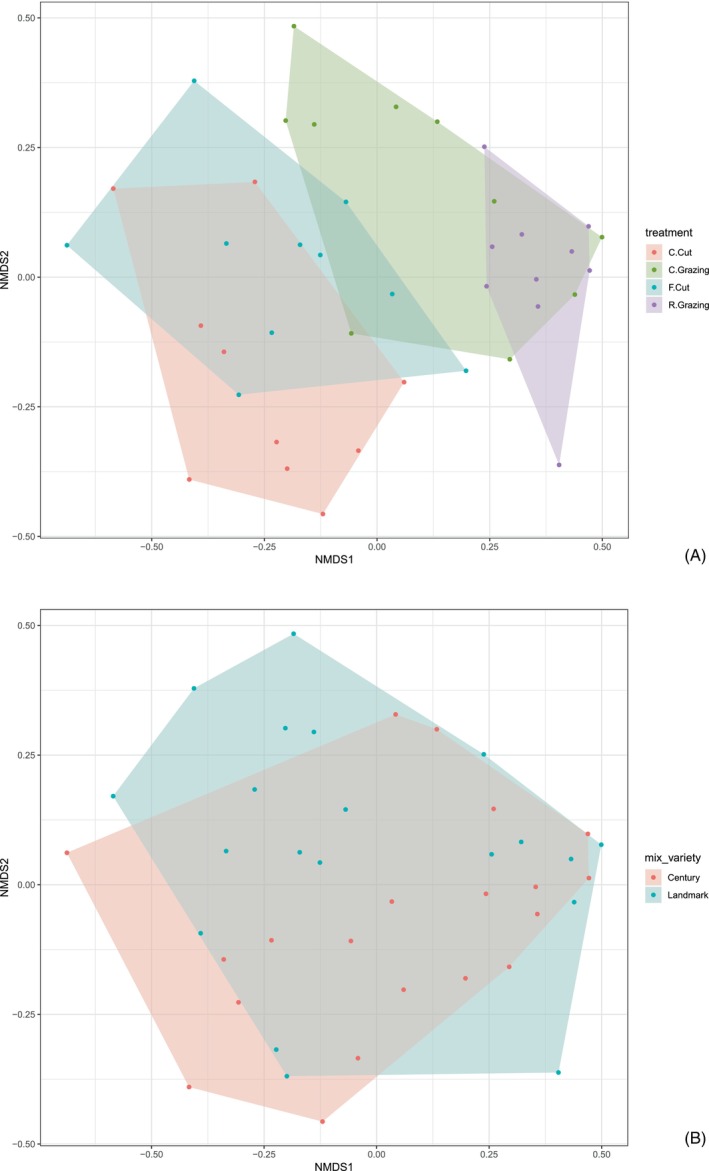
(A) Non‐metric multidimensional scaling (nMDS) ordination plot using Bray–Curtis dissimilarity on relative abundance values. The four management treatments present: Conservation cut (C.Cut), conservation grazing (C.Grazing), frequent cut (F.Cut) and rotational grazing (R.Grazing). Each point represents a sample. (B) NMDS ordination plot using Bray–Curtis dissimilarity on relative abundance values indicating clustering of samples by seed mixture (Century or Landmark) (B).

**FIGURE 5:**
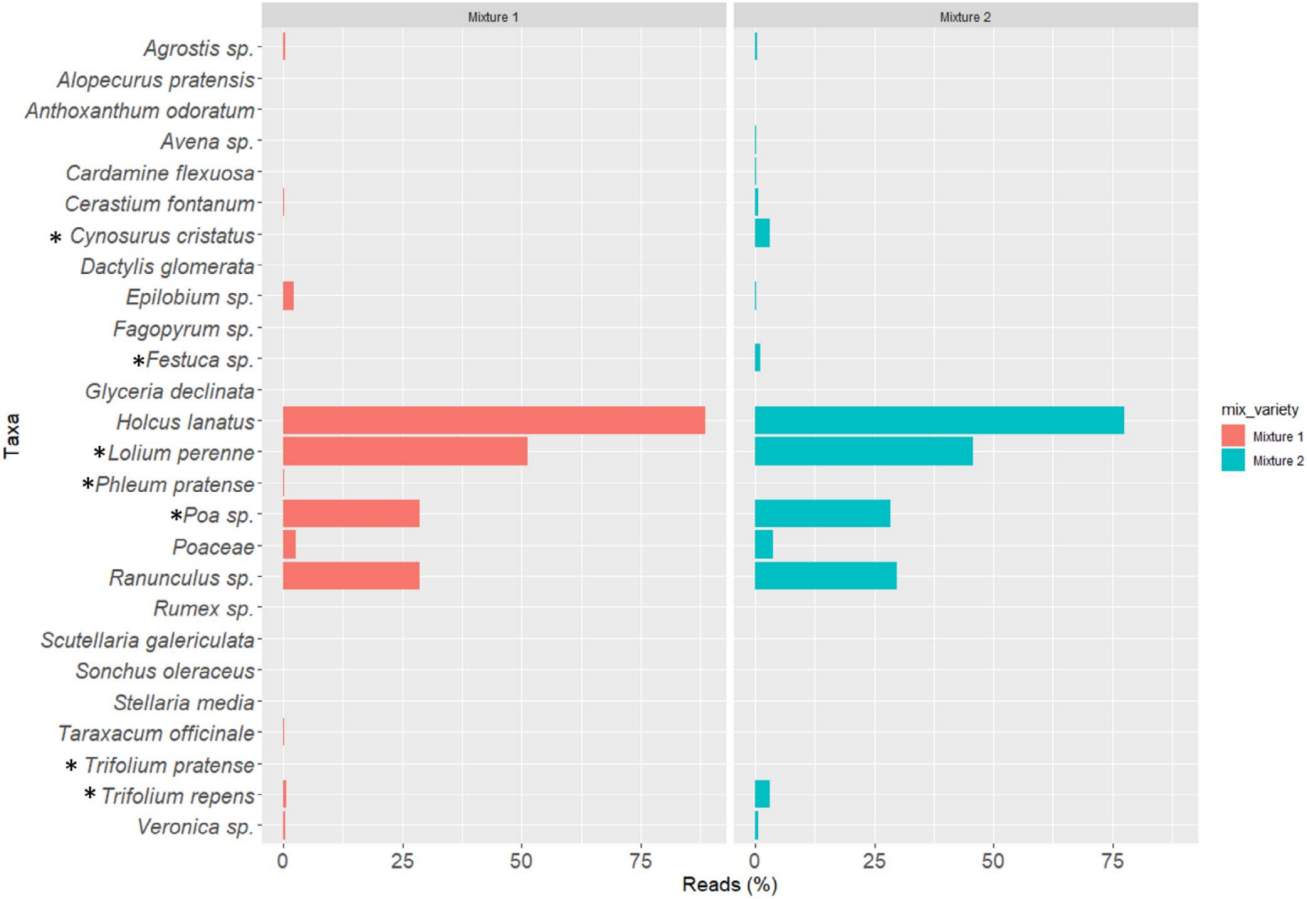
Taxa bar plot to show the relative abundance (%) of individual species present with each seed mixture variety, mixture 1 or mixture 2. (*) Indicates species that were originally sown in the test mixture varieties.

There were significant differences in botanical composition following different management treatments based on the DNA data (ANOVA test *p* = < 0.001). NMDS ordination plots of management type (treatment) showed similar community composition between the two cutting treatments and between the two grazing treatments, some overlap between frequent cutting and continuous grazing, but no similarity between either cutting treatment and rotational grazing (Figure 4A). Taxa that showed a significant contribution to the observed variation in the community composition included *Holcus lanatus* (*p* = 0.004), *Lolium perenne* (*p* = 0.009), *Phleum pratense* (*p* = 0.001), *Poa* spp. (*p* = 0.004) and *Ranunculus* spp. (*p* = 0.001). Some clustering was also observed by seed mixture varieties (Figure 4B). The species composition of the mixtures was significantly different (ANOVA *p* = 0.001), with two genera/species driving this, *Cynosurus cristatus* (*p* = 0.001) and *Festuca* sp. (*p* = 0.001; Figure 5).

**FIGURE 6:**
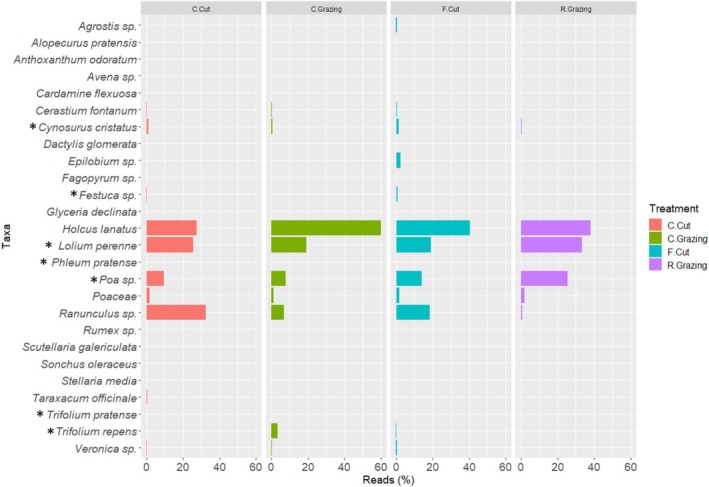
Taxa bar plot to show the relative abundance (%) of individual species present between each management treatment, conservation cut (C.Cut), conservation grazing (C.Grazing), frequent cut (F.Cut) and rotational grazing (R.Grazing). (*) Indicates species that were originally sown in the mixture varieties.

The family Poaceae was assigned to 67% of total reads, followed by Ranunculaceae (7%) and Fabaceae (6%), whilst the remaining 20% of reads were assigned to Caryophyllaceae, Asteraceae, Brassicaceae, Plantaginaceae and Polygonaceae. Within the family Poaceae, *H. lanatus* was the most dominant species present, followed by *L. perenne*. When looking at the species‐level identification of the combined marker set, both markers were only able to identify down to genus level for *Agrostis* spp., *Avena* spp., *Epilobium* spp., *Fagopyrum* spp., *Festuca* spp., *Poa* spp., *Ranunculus* spp., *Rumex* spp. and *Veronica* spp. (Figure 6).

There was a significant difference in plant composition between the treatment groups from the DNA data (LR_36_ = 289.3, *p* = 0.001) with a higher diversity in the cutting regimes compared to the grazing defoliation, as seen in the botanical survey dataset. There was a significant difference between frequent cutting versus continuous grazing and rotational grazing vs. frequent cutting (Table 2). There was no trend for the mixture to influence the plant diversity detected (*p* = 0.06).

**TABLE 2:** Pairwise comparisons between treatment groups to identify which specific groups are different from each other using Tukey's test and the metabarcoding dataset.

Treatment	*p* adj	
C.Grazing‐C.Cut	0.066	
F.Cut‐C.Cut	0.909	
R.Grazing‐C.Cut	0.107	
F.Cut‐C.Grazing	0.013	*
R.Grazing‐C.Graz	0.996	
R.Grazing‐F.Cut	0.024	*
Mix Variety	*p* adj	
Landmark‐Century	0.066	

* Indicates a significant result.

**FIGURE 7:**
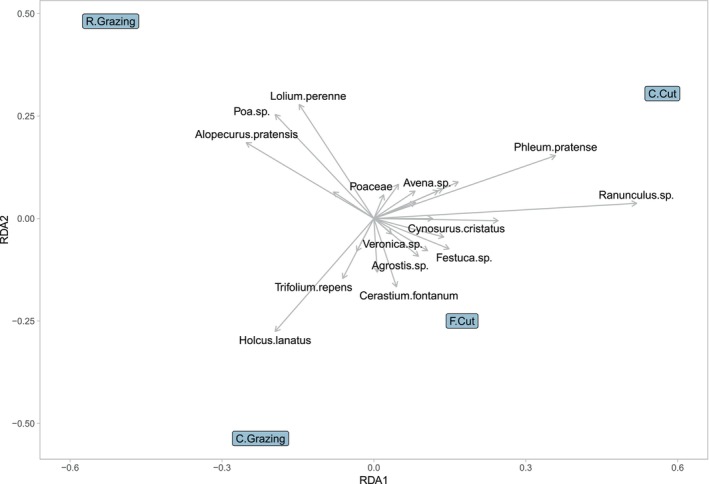
Ordination diagram representing the results of redundancy analysis (RDA) on the metabarcoding dataset displaying the most frequently found plant species within each treatment: rotational (R.) grazing, continuous (C.) grazing, frequent cutting (F.Cut), conservation cutting (C.Cut).

The RDA analysis examining the relationship between treatment and the species data explained a total of 26.5% of the variance (*R* ^2^ = 0.265). Additionally, the adjusted *R* ^2^ value, which takes into account both model complexity and the number of predictor variables, was 20.4% (adj.*R* ^2^ = 0.2041). These results suggest that the treatment variable has an influence on the species data, contributing to the observed variation. This was further supported by a permutation test. The RDA model with treatment as the predictor variable explained a significant amount of variance in the species data (Variance = 0.216, *F* = 4.334, *p* < 0.001). These results also suggest that the treatment variable has a statistically significant influence on the species data (*p* < 0.001), indicating that it plays a significant role in explaining the observed variation. Visualizing the RDA outcomes on a biplot (Figure 7) reveals the extent of the species’ impact on the ordination space (treatment), demonstrated by the length of the vectors (arrows). In rotational grazing, *L. perenne, Poa* spp. and *A. pratensis* dominate, whereas continuous grazing sees *H. lanatus* and *T. repens* as dominant. Regarding the cutting regimens, numerous species exhibit a positive correlation between the two treatments. Frequent cutting shows a dominance of *Cerastium fontanum, Agrostis* spp. and *Festuca* spp. Conversely, conservation cutting shows a greater influence of *P. pratense* and *Ranunculus* spp.

**FIGURE 8:**
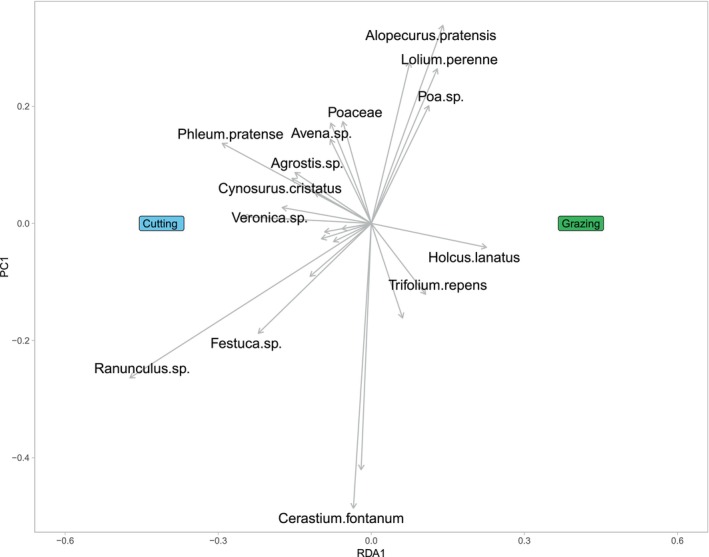
Ordination diagram representing the results of redundancy analysis (RDA) on the metabarcoding dataset displaying the most frequently found plant species within each defoliation regime: cutting and grazing.

The RDA analysis for defoliation type (cutting vs. grazing) only accounts for 11.25% of the observed variance in the species data (*R* ^2^ = 0.113) with the adjusted *R* ^2^ value (adj.*R* ^2^ = 0.089) explaining around 8.92% of the variance. Again, the permutation test results also suggest that the defoliation variables have a statistically significant influence on the species data (*p* < 0.001). The RDA plot (Figure 8) indicates that the cut swards are more species‐rich than the grazing management plots.

### 3.3 Summary of Comparison Between Methods

**FIGURE 9:**
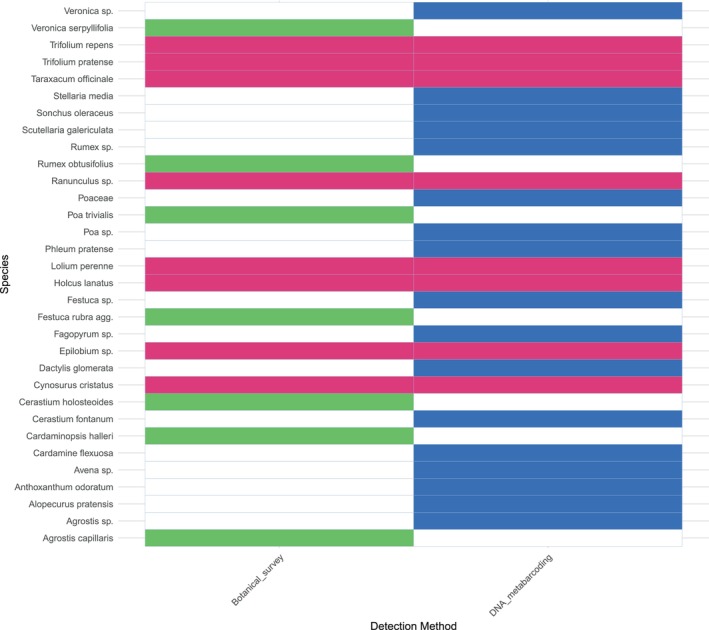
Heat table to show taxa identified in metabarcoding results (blue), botanical survey results (green) and taxa identified in both datasets (pink).

The plant community analysis from both the DNA metabarcoding and botanical survey data revealed that *Holcus lanatus* accounted for 42% of the overall species composition, followed by *Lolium perenne* (25%), *Ranunculus* sp. (15%), *Poa* sp. (7%) and *Trifolium repens* (4%). A Mantel test showed a significant correlation between the two datasets (*r* = 0.3169, *p* < 0.0001), indicating a degree of similarity in species composition. Overall, 25% of taxa overlapped between the DNA metabarcoding and botanical survey methods at the species level, highlighting areas of agreement (Figure 9). However, it should be noted that at the genus level, this overlap increases to 48%, demonstrating greater alignment between the two approaches when considering broader taxonomic classifications. In studies where species‐level identification is not essential, genus‐level resolution may provide a suitable and reliable alternative.

## 4 Discussion

### 4.1 Comparison of Approaches

Comparisons between the botanical survey and DNA metabarcoding datasets largely reveal similar findings on grassland community composition. However, the two approaches did identify different species as significant in driving community structure, and the botanical survey highlighted greater differences between the cutting and grazing treatments than the DNA data.

When comparing the two methodologies, DNA metabarcoding detected more species, including *Anthoxanthum odoratum* , which the botanical survey missed. This common grassland species typically flowers in May/June, and its absence in the survey may be due to the difficulty in identifying it vegetatively by July/August. This highlights how seasonal growth cycles can impact species detection in traditional surveys compared to DNA‐based methods. For several taxa, metabarcoding only provided identification at the genus level (*Agrostis, Avena, Fagopyrum, Festuca, Poa, Rumex, Veronica*), whereas the botanical survey demonstrated a greater ability to differentiate some of these genera further to the species level. Whilst these taxa can still correlate to each other e.g. *Agrostis capillaris* to *Agrostis* spp., there were two species included in the botanical survey results that suggest possible misidentification. *Cardaminopsis halleri* was recorded in the botanical survey while *Cardamine flexuosa* was identified via DNA metabarcoding. These two species visually look very similar, but *C. halleri* is not known to be present within the UK (confirmed via the Botanical Society of Britain & Ireland, and Plant Atlas 2020) suggesting there has been a misidentification. Additionally, *Cardaminopsis* is now recognized as a synonym for *Arabidopsis* according to Plants of the World Online (POWO), which further supports the likelihood of this misidentification. Furthermore, *C. flexuosa* is a species known to be present at the site; therefore, we would assume that metabarcoding has the correct assignment. Likewise, *Cerastium holostesoides* was identified in the botanical survey and *C. fontanum* in the metabarcoding; while both species belong to the same genus and family, *C. holostesoides* is a subspecies of *C. fontanum* . In this context, existing knowledge lends support to the likelihood of *C. fontanum* being the more probable species present.

Relative proportions of identified species also differed between the two methods. However, because there are numerous biases to consider that can affect the over‐ and under‐representation of species and lead to inaccurate quantification, the estimation of plant species identified via DNA metabarcoding can only be regarded as a semi‐quantitative measure (Zinger et al. 2019). For instance, the efficiency of DNA extraction can vary between species due to differences in cell wall composition, potentially leading to the underestimation of species with tough, fibrous tissues. PCR amplification bias may favor species with more abundant or easily amplifiable DNA, leading to a skewed representation in the final data. Overestimation can occur, particularly for species with high chloroplast concentrations, due to metabarcoding's ability to detect DNA from various organelles (chloroplast, mitochondrial and nuclear) within plant tissues, especially when chloroplast markers are used for amplification. (Taberlet et al. 2018; Pepeta et al. 2022).

Despite the differences in species identified and their proportions, both methodologies indicated that management treatment and seed mixture significantly impacted botanical composition, with outputs identifying the plant species contributing to these differences differing only slightly. Similarly, there was an overlap between the most abundant species present in DNA metabarcoding and botanical survey results, with *H. lanatus* and *L. perenne* common to both. Significant differences were observed in diversity between treatments for both methods, but the seed mixture had no impact. The differences between treatments were more pronounced for the botanical survey data, although the greatest change was linked to defoliation type (cutting vs. grazing) by both methods. It is evident from both methods that overall plant species diversity decreases among grazing treatments compared to cutting treatments, although the percentages of variations from the RDA analysis differ. It is well‐reported that intensive sheep grazing can decrease species richness in grassland ecosystems (Fraser et al. 2022). The extent of this impact is not uniform and can be influenced by several factors, such as the intensity and duration of a grazing period, specific management practices, resilience of the plant species that exist within a grassland (Boval 2012), and the composition of a sward if it contains highly preferential plant species to graze or not (Baur et al. 2007; Pavlů et al. 2021). Sheep can graze closely to the ground and will pick out preferred species when they are present, such as removing flowering plants while avoiding tall and rough grasses (Fraser et al. 2022). As the abundance of preferred species declines, this can lead to less nutritious, slower‐growing species dominating, reducing the overall species richness of an area. This transformation can have further detrimental effects driving shifts in sward composition, loss of soil nutrients and soil compaction leading to an overall decline in the biodiversity of a grassland habitat (Fraser et al. 2022). Generally, the grazing‐only treatments were dominated by the grasses *H. lanatus* and *L. perenne* , with the results for the continuously grazed treatments also indicating an increase in cover of *T. repens* . This finding is somewhat surprising as sheep usually select *Trifolium* spp. in preference to grass, but it may reflect the clover becoming very small‐leaved to avoid being grazed. Further analysis is underway to determine the consistency of this finding across sites and seasons.

A study conducted by Mattia et al. (2012) demonstrates a case for the advantages of molecular DNA barcoding over traditional morphology‐based techniques with vegetation surveys. Their research demonstrated that DNA barcoding not only delivers more precise species identification but also streamlines the entire process in terms of time and cost (van der Heyde et al. 2022). Mattia et al. (2012) highlight the importance of requiring a well‐established, study‐specific reference sequence library when employing a multi‐marker approach for effective DNA barcoding. However, the study also describes certain limitations, including an incapability to detect morphological characteristics, assess species cover and evaluate vegetation quality, all of which can be influenced by environmental factors. In this study, it was concluded that misidentification was likely the cause of the discrepancies in the identification of *C. helleri* in the botanical survey compared to *C. flexuosa* in the sequence results. Similarly, Mattia et al. (2012) found certain species were solely detected through barcoding, potentially because they were overlooked or misidentified in vegetation surveys. This finding emphasizes that human error and identification skills are significant limitations of conventional survey methods (Mattia et al., 2012). It has been reported that DNA‐based approaches to monitoring plant communities have the potential to answer large knowledge gaps in plant biodiversity studies, particularly for species requiring urgent conservation attention as traditional methods, such as botanical surveys, have often struggled to capture the full extent of biodiversity, especially in species‐rich communities (Johnson et al. 2023). DNA‐based techniques have proven to be as effective as or even more so than traditional methods. However, combining methods can improve species detection and resolution (Banerjee et al. 2022).

Despite the rapid advancement of DNA techniques for the identification and differentiation of species, there are still several challenges to overcome, such as the incompleteness of global DNA reference sequence libraries, the ongoing development of standard universal primers and the need for standardized protocols. Nonetheless, widely used standard barcodes for plant applications (including *rbc*L, *trn*L, *mat*k and ITS2) have been proven to work well in many metabarcoding studies (Kress and Erickson 2007; Hollingsworth et al. 2009; De Mattia et al. 2012; De Vere et al. 2012, 2015; Fahner et al. 2016; Kress 2017; Leontidou et al. 2021). It is important to note that no single DNA marker is capable of completely differentiating between all species at the highest taxonomic resolution (Kress 2017). Because of this, numerous studies suggest the use of a multi‐marker approach (as demonstrated in this study) to address key research questions for the desired taxonomic resolution (Hollingsworth et al. 2009, 2011; Bell et al. 2017; Jones et al. 2021).

### 4.2 Methodological Considerations

As previously mentioned, one of the crucial steps required to further advance DNA‐based methods is the establishment of a comprehensive global plant DNA barcode library for universal use. Significant efforts have been made to create such libraries, as demonstrated by De Vere et al. (2012), which gives a national database covering the native flowering plants and conifers of Wales, Jones et al. (2021), which gives a national DNA barcoding database for all the native conifers and flowering plants in the United Kingdom, and Kuzmina et al. (2017), which report the building of a DNA barcode library for Canadian vascular plants using DNA from herbarium specimens. The continued development of comprehensive libraries is of great importance for biodiversity research and conservation efforts. Verified references for DNA metabarcoding can aid in species identification, the ability to monitor changes in plant communities over time and contribute to a wider understanding of plant biodiversity (Jones et al. 2021).

The ability to quantify species abundance via DNA metabarcoding poses several challenges in achieving accurate results. Variability in DNA amplification can occur from the initial PCR stages due to primer bias or the quality and concentration of extracted DNA starting material. This can lead to the over‐amplification of some taxa and the under‐representation of others, ultimately resulting in quantification inaccuracies. Additionally, differences in DNA content across species may also influence the number of reads assigned to each species, as species with higher DNA content can disproportionately dominate the sequencing output. During laboratory processes, there is scope for human error, which can introduce contaminants leading to incorrect amplification (Alberdi et al. 2019). To address this concern, the current study utilized a previously optimized PCR protocol (De Vere et al. 2012), though primer choice remains a factor influencing quantification accuracy. The choice of primers can bias the results by favoring the amplification of some taxa over others. However, using well‐established primers (such as those used here for ITS2 and *rbc*L), which have a successful track record (Hollingsworth et al. 2009, 2011; De Vere et al. 2012, 2015; Fahner et al. 2016; Braukmann et al. 2017; Kuzmina et al. 2017; van der Heyde et al. 2020; Jones et al. 2021), can reduce these concerns. Including trnL primers might further enhance results and taxonomic assignments, as trnL has been reported to outperform rbcL as a DNA metabarcoding marker. For example, trnL has been shown to more accurately identify plant species in dietary metabarcoding studies (Mallott et al. 2018). This advantage may be attributed to the shorter marker length of trnL, which is more suitable for working with degraded DNA, such as that found in fecal material, enabling more successful amplification and sequencing of partially digested plant DNA (Hollingsworth et al. 2011; Moorhouse‐Gann et al. 2018; Taberlet et al. 2018).

The pre‐processing of sequence data through bioinformatic pipelines introduces another layer of complexity and subjectivity with regard to read depth, filtering, quality trimming and taxon assignment, as often this is decided based on an individual's interpretation (Hollingsworth et al. 2011; Zinger et al. 2019; Luo et al. 2023; Hakimzadeh et al. 2024). To mitigate this issue, it would be beneficial in future studies to consider processing sequence data through multiple bioinformatic pipelines to compare results and ensure data reliability (Hakimzadeh et al. 2024). It is evident from the literature that further development of user‐friendly bioinformatics tools is required to standardize bioinformatic processes (Deiner et al. 2017; Ruppert et al. 2019; Banerjee et al. 2022). Luo et al. (2023) advocate the use of spike‐in standards as controls to be included in studies, which involves a known quantity of a specific DNA sample to test the accuracy, reproducibility and reliability of sequence data (Harrison et al. 2021; Luo et al. 2023). This concept is something that should be more widely considered for future applications and studies utilizing DNA metabarcoding.

For this study, several mitigations were implemented based on suggestions from the literature, including using multiple markers (*rbc*L & ITS2), conducting quality control checks with both positive and negative controls and implementing the use of an accurate curated reference library. Nevertheless, challenges related to quantification and discrepancies in taxon assignment still arose, highlighting the importance of continued research and development to advance the precision of DNA metabarcoding for use in the field. What this study does demonstrate is that there are notable advantages of DNA metabarcoding over traditional botanical surveys. Unlike traditional methods, DNA metabarcoding does not rely on the presence of identifiable plant features such as flowers, making it possible to conduct sampling throughout the year. This is particularly advantageous in situations where plants may not be fully developed, such as newly germinated species or those that have just started growing after dormancy, which can be challenging for even experienced botanists to identify based on leaves or roots alone. Additionally, metabarcoding only requires a small amount of plant tissue for processing, further reducing the need for whole herbarium samples. Finally, while a thorough reference database can streamline the identification process, botanical identification skills remain valuable. Prior botanical knowledge of a site or system can significantly enhance computer‐assigned identifications by reducing false positives or erroneous IDs, especially when resolving difficult or uncommon taxa. Examining results in conjunction with expert insight helps ensure greater accuracy, particularly in complex ecological systems (Banerjee et al. 2022). DNA metabarcoding is a promising complementary tool to traditional methods for monitoring grasslands, although further research is essential to refine the quantification of DNA metabarcoding, enabling its integration into future research as a supplementary tool.

Future work on DNA metabarcoding for mixed‐species pastures should focus on expanding and refining reference libraries (Johnson et al. 2023; Kestel 2022), specifically for pasture species to improve identification accuracy. Optimizing sampling and DNA extraction protocols (van der Heyde et al. 2020) tailored to pasture environments is also crucial. Integrating DNA metabarcoding with traditional survey methods could enhance species assessments (Hassan 2022), while longitudinal studies could track temporal changes in species composition under various management practices. Our results demonstrate that species diversity was higher in cut plots compared to grazed plots, aligning with previous studies that suggest grazing often exerts selective pressure on palatable species such as clovers, reducing overall diversity, while cutting can provide more uniform defoliation, encouraging species richness (Fraser et al. 2022). However, discrepancies in taxonomic resolution between methods and the semi‐quantitative nature of DNA metabarcoding highlight the need for further refinement to achieve consistent outcomes across study systems. Research should also explore the economic feasibility of using DNA metabarcoding for widespread adoption in sustainable grazing systems, including its potential for informing pasture management and restoration (Kestel 2022).

## 5 Conclusion

In summary, both DNA metabarcoding and botanical surveys provided valuable insights into species composition and diversity. While there are some differences in the number of taxa identified and the most abundant species, overall, the findings generated were similar. The results of this study demonstrate the potential of DNA metabarcoding as a reliable and efficient approach for characterizing mixed‐species pastures. By offering a scalable alternative to traditional methods, DNA barcoding of plant communities presents exciting opportunities for biodiversity research, particularly as advancements in technology, methodologies and bioinformatics continue to evolve. This study highlights how DNA metabarcoding can streamline plant community assessments, reduce dependence on taxonomic expertise and complement traditional survey techniques. These benefits have practical implications for grassland management, enabling more accurate and timely tracking of species diversity and composition, which are essential for developing sustainable grazing practices and effective conservation strategies. Continued research will further enhance the precision and reliability of DNA metabarcoding, advancing our understanding of plant communities, their interactions with herbivores and their role in addressing global biodiversity challenges.

## Author Contributions

**Hannah Vallin:** conceptualization (equal), investigation (equal), data curation (equal), formal analysis (lead), writing – original draft (lead). **Helen Hipperson:** formal analysis (supporting), writing – review and editing (equal). **Jan Titěra:** funding acquisition (equal), investigation (equal), data curation (equal). **Laura Jones:** formal analysis (supporting), writing – review and editing (equal). **Mariecia Fraser:** conceptualization (equal), funding acquisition (equal), writing – review and editing (equal).

## Conflicts of Interest

The authors declare no conflicts of interest.

## Data Availability

All data supporting the findings of this study, including the trimmed plant DNA sequences, are publicly available in the Dryad Digital Repository (DOI): https://doi.org/10.5061/dryad.dv41ns27x. The dataset contains paired‐end sequences for ITS2 and rbcL regions, prepared to facilitate replication and further analyses of plant community composition.
